# Protein Bioaccessibility of Unfermented Chickpea Milk and *Lactiplantibacillus plantarum* Fermented Chickpea Milk as Evaluated by *In Vitro* Gastrointestinal Digestion

**DOI:** 10.1002/fsn3.70941

**Published:** 2025-09-21

**Authors:** Wen Li, Tao Wang

**Affiliations:** ^1^ Jiangsu Province Engineering Research Center for Food Biological Transformation and Safety Detection Xuzhou University of Technology Xuzhou P. R. China

**Keywords:** bioaccessibility, chickpea protein, in vitro gastrointestinal digestion, lactic acid bacteria, proteolysis

## Abstract

In this study, *In vitro* gastrointestinal digestion (GIS) was utilized to investigate the protein bioaccessibility of chickpea milk nonfermented and fermented with *Lactiplantibacillus plantarum*. The soluble protein, peptide, and free amino acid contents; protein degradation; peptide profiles; degree of hydrolysis (DH); and particle size were investigated before digestion and during the oral, gastric, and intestinal digestion phases. Results showed that the soluble protein content of *L. plantarum* fermented chickpea milk (LFCM) was lower than that of unfermented chickpea milk (UFCM) after digestion. Proteolysis resulted in increased release of peptides and free amino acids. Smaller peptides gradually increased, and LFCM reached a higher peptide content after digestion. The electrophoretic profiles also indicated fast proteolysis. High‐performance liquid chromatography (HPLC) presented more peaks in the digested LFCM profile than that of UFCM after digestion. Moreover, LFCM showed a higher degree of hydrolysis (DH) and a smaller particle size at the end of in vitro GIS. These data suggested that chickpea milk fermented with *L. plantarum* and the subsequent digestion was more effective in proteolysis, thus showing more potential protein bioaccessibility than the digestion of chickpea milk alone.

## Graphical abstract

The protein bioaccessibility of unfermented chickpea milk (UFCM) and *Lactiplantibacillus plantarum*‐fermented chickpea milk (LFCM) was evaluated following in vitro gastrointestinal digestion (GIS). Electrophoretic analysis revealed rapid proteolysis during GIS, leading to enhanced release of peptides and free amino acids. Compared to UFCM, LFCM exhibited a smaller mean particle size (D [4,3]), a higher concentration of peptides, and slight differences in peptide profilesafter digestion. These findings suggest that the combination of fermentation and digestion may enhance chickpea protein bioaccessibility more effectively than digestion alone.

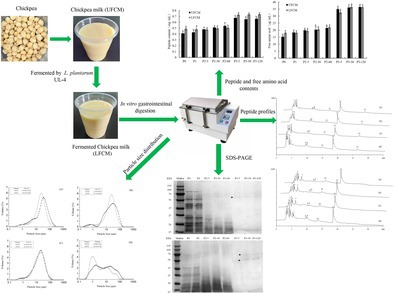

**Abbreviations**
DH: degree of hydrolysis
FAAs: free amino acids
GIS: *in vitro* gastrointestinal digestion
HPLC: high performance liquid chromatography
*L. plantarum*
: *Lactiplantibacillus plantarum*
LAB: lactic acid bacteria
LFCM: *L. plantarum* fermented chickpea milk
MRS: in de Man, Rogosa, and Sharpe
PBS: phosphate‐buffered saline
RT: retention times
SDS: sodium dodecyl sulfate
SDS‐PAGE: sodium dodecyl sulfate–polyacrylamide gel electrophoresis
UFCM: unfermented chickpea milk

## 1 Introduction

Chickpea (*Cicer arietinum L*.) is one of the most widely consumed and oldest beans; it is enriched in proteins (21%–25%), minerals, and fiber. In addition, high levels of amylose and resistant starch in chickpeas may reduce the risk of hypertension and type II diabetes (Zhang, Tang, et al. 2022). Chickpea milk, a water‐soluble extract based on the process of maceration, grinding, and filtration of chickpeas (Munekata et al. 2020), is often regarded as a prospective plant‐based drink substitute for milk. Compared to dairy products, chickpea milk offers improved nutritional quality, greater food safety, lower fat content, fewer allergens, and reduced risk of lactose intolerance (Sethi et al. 2016; Zhang, Tang, et al. 2022). Moreover, chickpea milk could also be a potential substitute for soymilk for its organoleptic and nutritional properties (Wang et al. 2018). Microbial fermentation is a well‐established way to improve the nutritional quality of plant‐based milk alternatives (Tangyu et al. 2019). Fermented chickpea milk is a yogurt‐like type of drink fermented by lactic acid bacteria (LAB) (Zhang, Zhang, et al. 2022), because of its similar high content in protein, which can be coagulated during LAB fermentation. As a probiotics‐containing fermented legume drink, it has been confirmed to possess improved flavor, nutritional, and functional value, and higher digestibility of indigestible sugars by former studies (Meng et al. 2023; Tangyu et al. 2019; Tangyu et al. 2021; Zhang, Tang, et al. 2022; Zhang, Tian, et al. 2022). Therefore, the development of fermented chickpea milk with improved nutritional and health benefits has attracted increasing attention.

However, studying the composition of a certain food is not enough to predict its health benefits. The bioaccessibility and bioavailability of food components are crucial information. According to Rodrigues et al. (2022), bioaccessibility represents the fraction of a nutrient that becomes available for absorption after being released from the food matrix through digestion. Bioaccessibility studies are important for people to make nutrition plans and provide the closest requirements. However, bioaccessibility studies have disadvantages in terms of time, procedures, and cost (Hur et al. 2011). *In vitro* gastrointestinal digestion, which simulates the physiological conditions of the oral, gastric, and small intestinal phases of digestion, is an effective strategy to predict outcomes of in vivo gastrointestinal tract digestion since it is rapid, safe, and does not have the ethical restrictions existing in vivo methods (Bohn et al. 2018). The protocol has been widely used to study protein digestion in many kinds of legume matrices and to evaluate the effect of different treatments on the protein bioaccessibility of legumes. Ayala‐Rodríguez et al. (2022) investigated the *in vitro* nutritional characteristics of four fava bean protein flours, demonstrating that heat treatment notably enhanced protein digestibility. Polyphenol‐protein concentrate showed the highest bioaccessibility; moreover, bioaccessible low molecular weight peptides (< 15 kDa) were produced after *in vitro* digestion for all the flours. Another recent study assessed the *in vitro* protein bioavailability of plant‐based proteins from pea, soy, and fava bean ingredients via the degree of protein hydrolysis (DH). The average DH after *in vitro* digestion was similar for all ingredients (Auer et al. 2024). Delgado‐Andrade et al. reported that biscuits containing chickpea exhibited increased protein hydrolysis and higher amino acid levels following digestion when compared to conventional shortbread biscuits (Delgado‐Andrade et al. 2024). Fermentation has also been successfully used as a pre‐treatment of different legume flours for the improvement of protein digestibility (De Pasquale et al. 2020). Zhou et al. examined the impact of solid‐state fermentation using *P. pentosaceus* strains on chickpeas and observed that fermented samples exhibited enhanced protein digestibility, along with a greater abundance of small peptides and bioactive peptides during *in vitro* digestion (Zhou et al. 2025). To our knowledge, limited data are available regarding the systematic comparison of *in vitro* GIS on the protein bioaccessibility of chickpea milk before and after LAB fermentation.

This research aimed to evaluate the effect of *in vitro* gastrointestinal digestion on the protein bioaccessibility of chickpea milk nonfermented and fermented with *L. plantarum* UL‐4. The soluble protein, peptide, and free amino acid contents were determined, and degradation of protein, peptide profiles, degree of hydrolysis, and particle size were also investigated. Results of the study will be helpful in understanding how LAB fermentation affects the bioaccessibility of chickpea protein during *in vitro* GIS, and thus will be expected to provide information in designing novel chickpea probiotic products.

## 2 Materials and Methods

### 2.1 Materials

Chickpeas (*Kabuli*) were obtained from a supermarket in Xinjiang Uygur Autonomous Region, China. Enzymes and reagents, including α‐amylase (A1031), pepsin (P7000), bile acid (B8631), pancreatin (P3292), and sodium dodecyl sulfate (SDS), were sourced from Sigma‐Aldrich Chemical Co. (St. Louis, MO, USA). The protein marker (15–150 kDa) was acquired from Sangon Biotech Co. (Shanghai, China), while HPLC‐grade acetonitrile and trifluoroacetic acid were supplied by Merck (Darmstadt, Germany). All other analytical‐grade chemicals were purchased from Sinopharm Chemical Reagent Co. Ltd. (Shanghai, China).

### 2.2 Bacterial Strains and Culture Conditions

The experiment utilized *L. plantarum* UL‐4, a strain originally isolated from fermented pickles and subsequently improved through compound mutagenesis. Prior to use, the strain was activated by two sequential subcultures in de Man, Rogosa, and Sharpe (MRS) broth (Merck, Darmstadt, Germany) at 37°C for 12 h. Following incubation, bacterial cells were harvested via centrifugation (8000 × g, 4°C, 15 min) and subjected to two washing cycles with sterile physiological saline.

### 2.3 Preparation of Fermented Chickpea Milk Using *L. plantarum* UL‐4

Chickpeas were thoroughly rinsed and soaked in distilled water at room temperature for 12 h. Following soaking, water was discarded, and the soaked chickpeas were boiled in 10 times (w/v) distilled water for 15 min using an electric pot (WH2202S, Midea, Shunde, China). Subsequently, the boiled chickpeas were processed by continuous wet‐milling for 5 min with a homogenizer (MJ‐WBL2501B, Midea, Shunde, China) and filtered with a double‐layered cheesecloth. The filtered chickpea milk was then sterilized at 108°C for 15 min. After cooling, the chickpea milk was inoculated with *L. plantarum* UL‐4 (10^7^ CFU/mL initial count) and incubated at 37°C for 48 h (Li et al. 2016). The resulting fermented product was then stored at 4°C for 12 h to allow post‐maturation.

### 2.4 *In Vitro* Gastrointestinal Digestion (GIS)

In vitro simulation of salivary, gastric, and intestinal digestion was conducted according to a previously reported protocol by Shim et al. (2010) with some modifications, and the ratio of food to digestive juice was 2.5: 1.0: 1.5: 1.0: 1.0 (food: saliva: gastric juice: pancreatic juice: bile acid) to simulate human physiological conditions. During the digestion process, the pH value was adjusted with NaOH or HCl (4 M). To simulate buccal chewing digestion, 25 mL of unfermented chickpea milk (UFCM) or *L. plantarum* fermented chickpea milk (LFCM) was added with 10 mL of α‐amylase solution (w/v, 0.2 mg α‐amylase in 20 mM phosphate buffer, pH 7.0) and incubated at 37°C in a shaking water bath (SWB series, HerryTech, Shanghai, China) at 55 r/min for 3 min. For gastric digestion, the pH was adjusted to 2.0, followed by the addition of 15 mL gastric juice (3.2 mg pepsin in 0.1 M HCl), and digestion proceeded at 37°C, 55 r/min for 60 min. Subsequently, intestinal digestion was simulated by adjusting the pH to 7.0 and adding 10 mL of bile acids and 10 mL of pancreatic fluid (0.4 mg enzyme in 10 mM phosphate buffer, pH 7.0), with agitation at 150 r/min for 120 min. Samples were collected before digestion (P0), after buccal digestion (P1), at 5, 30, and 60 min of gastric digestion (P2‐5, P2‐30, P2‐60), and at 5, 30, and 120 min of intestinal digestion (P3‐5, P2‐30, P2‐120), respectively. After each stage, samples were immediately heat‐inactivated in a boiling water bath (5 min). Except for particle size analysis, all samples were centrifuged (10,000 × g, 20 min, 4°C), and the supernatant was stored at −20°C.

### 2.5 Soluble Protein Content

The soluble protein content in the digestion supernatants was measured using the Bradford assay (Bradford 1976), with bovine serum albumin (BSA) as the standard. The soluble protein content was expressed as μg protein per mL chickpea milk (μg/mL).

### 2.6 Electrophoresis

The degradation of chickpea proteins during digestion was analyzed using sodium dodecyl sulfate‐polyacrylamide gel electrophoresis (SDS‐PAGE) with a 15% separating gel and 4% stacking gel. Electrophoresis was performed on a Miniprotein 3 unit (Bio‐Rad Laboratories Inc., Hercules, CA, USA) under constant voltage (60 V for stacking, 120 V for separating). Following separation, the gels were stained with 0.1% (w/v) Coomassie Brilliant Blue R‐250 and scanned using an Image Scanner III (GE Healthcare Biosciences, Uppsala, Sweden). Protein molecular weights were estimated using a prestained marker (15–130 kDa; C610011, Sangon Biotech, China), and band intensities were quantified with Quantity One software, version 4.6.2 (Bio‐Rad Laboratories Inc., Hercules, CA, USA).

### 2.7 Peptide Content and Total Free Amino Acids Content

The peptide contents of the digestion supernatants were determined in accordance with the o‐phthaldialdehyde (OPA) assay (Rui, Fu, et al. 2016). At each sampling interval, supernatants were filtered through 10 kDa molecular weight cutoff membranes (Millipore, USA). The filtrate (50 μL) was collected and added to 2 mL OPA solution. The OPA working solution (100 mL total volume) was prepared by combining 200 μL β‐mercaptoethanol, 80 mg OPA (in 2 mL methanol), 5.0 mL 20% (w/w) SDS, and 50 mL 100 mM borax buffer. Following a 2 min room temperature incubation, absorbance was measured at 340 nm (T600AS, Beijing Purkinje General Instrument Co. LTD, China). The total free amino acid content was quantified using the method reported by Hu et al. (2007) with glycine as a standard (Hu et al. 2007). The peptide content and free amino acid content were both expressed as mg/mL of chickpea milk.

### 2.8 Peptide Analysis by High Performance Liquid Chromatography (HPLC)

Peptide analysis was investigated following a previously established protocol (Li and Wang 2021). Chromatography employed a ZORBAX Eclipse Plus C18 column (4.6 × 250 mm, 5 μm) with 0.1% trifluoroacetic acid [TFA] in water (A) and acetonitrile (B) as mobile phase components. Samples (20 μL) were eluted with the following solvent gradients: from 0 to 2 min, 95%–95% A; from 2 to 7 min, 95%–90% A; from 7 to 10 min, 90%–85% A; from 10 to 20 min, 85%–80% A; from 20 to 30 min, 80%–70% A; from 30 to 40 min, 70%–0% A; and from 40 to 45 min, 0%–95% A. The monitor wavelength was set at 280 nm by using a DAD detector (GB15B), and the flow rate and column temperature were set at 0.8 mL/min and 25°C, respectively.

### 2.9 Degree of Hydrolysis

The degree of hydrolysis was quantified using the OPA method (Church et al. 1983) with L‐serine as the standard. Briefly, 400 μL supernatant was mixed with 3 mL of OPA reagent, incubated for 2 min at room temperature, and the absorbance was measured at 340 nm.

### 2.10 Particle Size Distribution

The particle size distribution of samples at each digestion stage (P0, P1, P2‐60, and P3‐120) was measured by a light scattering instrument (BT‐9300st, Dandong Baxter Instrument Co. LTD, Shandong, China). Refractive indices of 1.56 and 1.33 were used for the chickpea protein dispersions and dispersing phase (water), respectively. The ultrasonic dispersion was set for 2 min, and the speed of the circulating pump was set at 1600 r/min. Particle size values were measured as D [4,3].

### 2.11 Statistical Analysis

All analyses were performed in triplicate. Data are presented as means, with statistical significance (*p* < 0.05) assessed by independent Student's t‐test using IBM SPSS software.

## 3 Results and Discussion

### 3.1 Soluble Protein Contents During *In Vitro* GIS for Unfermented Chickpea Milk (UFCM) and *L. plantarum* Fermented Chickpea Milk (LFCM)

**FIGURE 1:**
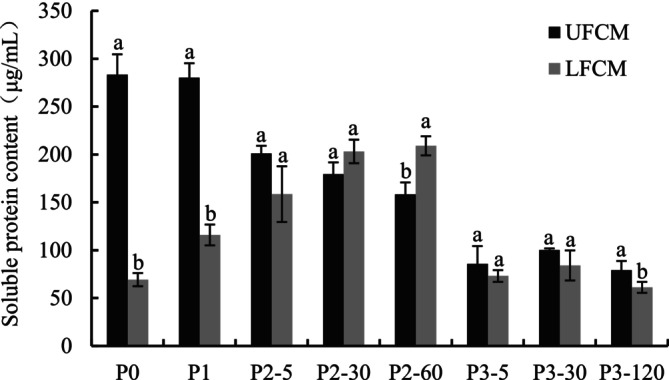
Soluble protein contents of unfermented chickpea milk (UFCM) and *L. plantarum* fermented chickpea milk (LFCM) subjected to *in vitro* gastrointestinal digestion (GIS). Within the same digestion phase, different lowercase letters indicate significant differences (*p* < 0.05). P0: Before the GIS; P1: After buccal digestion; P2‐5, P2‐30, P2‐60: Samples taken at 5 min, 30 min, and 60 min of gastric digestion; P3‐5, P3‐30, P3‐120: Samples taken at 5 min, 30 min, and 120 min of intestinal digestion. Data are expressed as mean ± SD of triplicates.

The changes in soluble protein content in UFCM and LFCM at different stages of the digestion process are shown in Figure 1. Before digestion (P0), the soluble protein content in LFCM was much lower than UFCM; this was in accordance with the results reported by Rui, Fu, et al. (2016). The reduced soluble protein content in fermented chickpea milk (LFCM) likely resulted from acid‐induced gelation during LAB fermentation, where acids neutralized negative protein complexes (Guo and Ono 2005). No change occurred in UFCM, while LFCM showed a slight protein solubilization increase after the buccal digestion (P1) phase. During the gastric digestion (P2) and intestinal digestion (P3) phases, the soluble protein content in UFCM decreased with the extension of time. As for LFCM, whose soluble protein content increased during the gastric phase and decreased during the intestinal phase. This decrease likely reflected extensive proteolytic degradation of larger proteins into smaller peptides and amino acids, which are undetectable by the Coomassie brilliant blue method (Compton and Jones 1985). During the P2 phase, due to the continuous mechanical shaking, the hydrolytic action of pepsin, and the acidic pH, the trapped proteins were released from the matrix to the solution; thus, there was an apparent increase in LFCM. By the end of the gastric phase, soluble protein contents increased sharply to 209.05 μg/mL in LFCM. A similar result was reported by Rinaldi et al. (2014), that soluble protein contents for yogurt digest were higher after simulated gastric digestion. At the end of gastrointestinal digestion, the soluble protein content was 79.23 μg/mL in UFCM, which was a little higher than that in the LFCM, namely 61.20 μg/mL. Overall, this indicates that the released soluble protein is continuously decomposed and utilized under the action of digestive enzymes.

### 3.2 Electrophoretic Analysis

**FIGURE 2:**
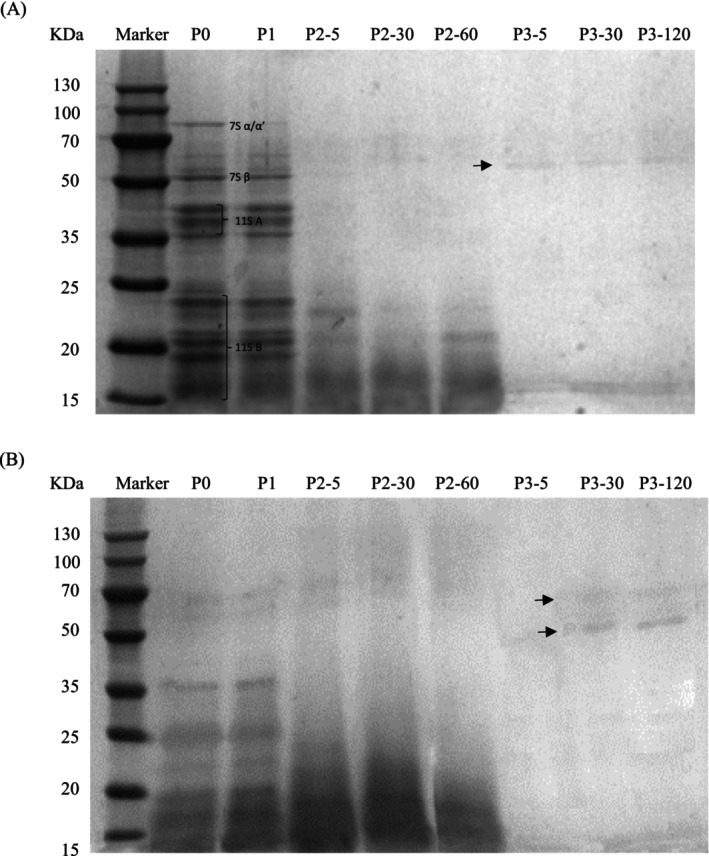
Sodium dodecyl sulfate–polyacrylamide gel electrophoresis (SDS‐PAGE) of protein profiles at different phases of *in vitro* GIS. P0: Before the GIS; P1: After buccal digestion; P2‐5, P2‐30, P2‐60: Samples taken at 5 min, 30 min, and 60 min of gastric digestion; P3‐5, P3‐30, P3‐120: Samples taken at 5 min, 30 min, and 120 min of intestinal digestion. Samples were resolved on a 15% separating gel. 7S α/α’ and 7S β subunits: Subunits of β‐conglycinin; 11S A and 11S B subunits: Acidic and basic subunits of glycinin.

Electrophoresis was further performed to determine the changes in the protein profile of UFCM and LFCM during the *in vitro* GIS digestion. Results are presented in Figure 2. The most intensive protein bands with MWs between 17 and 85 kDa were detected in the supernatant of UFCM at the P0 phase (lane P0, Figure 2A). These bands corresponded to the α'/α (~85 kDa) and β (~52 kDa) subunits of 7S conglycinin and the acidic (~36–42 kDa) and basic (~17–25 kDa) units of 11S glycinin (Jenkins and Beaudoin 2004). As to the supernatant of LFCM, a limited number of bands with molecular mass (MM) of 68, 60, 36, 25, 22, 20, and 19 kDa were presented at the P0 phase (lane P0, Figure 2B), which was in accordance with the earlier finding of lower soluble protein content compared with UFCM. Protein profiles of both samples were almost unchanged after simulated buccal digestion (lane P1, Figure 2A,B), probably because there was no effect of proteases in this digestion phase.

However, gastric digestion (P2) induced dramatic decreases of visible bands for both samples from UFCM and LFCM gels. UFCM sample presented a depletion of 7S conglycinin and the acidic units of 11S glycinin (lane P2‐5, Figure 2A), which might stand for rapid degradation of 7S globulin and acidic units of 11S glycinin in UFCM gel. Bands ranging from 17–25 kDa, corresponding to basic units of 11S glycinin, seemed to be resistant to the peptic digestion and were visible at P2‐5 (lane P2‐5, Figure 2A); after that, they were partially hydrolyzed but still visible at P2‐60 (lanes P2‐30, P2‐60, Figure 2A). In terms of LFCM, gastric digestion resulted in the depletion of most bands, except for bands ranging from 15–23 kDa, which presented increased band intensity at P2‐5 and P2‐30 (lane P2‐5, 30, Figure 2B) and were partially hydrolyzed at the end of peptic digestion (lane P2‐60, Figure 2B), which might indicate a rapid degradation of larger protein during the gastric digestion phase.

Duodenal digestion (P3) resulted in the disappearance of most bands in UFCM and LFCM gels. The faint band at MM of 18 kDa remained in the UFCM gel until the end of the P3 phase (lane P3‐5, P3‐30, P3‐120, Figure 2A). The LFCM gel showed no presence of a visible band at P3‐5 (lane P3‐5, Figure 2B). Proteins might have been degraded further into lower molecular mass peptides that could not be detected by SDS‐PAGE. On the other hand, duodenal digestion induced increases in faint visible bands for both samples from UFCM and LFCM gels. The band at MM of 60 kDa for a sample of UFCM, and bands at 70 and 55 kDa for a sample of LFCM (Bands were pointed by arrows at lane P3‐5, Figure 2A and lane P3‐30, Figure 2B), these new bands were stable along with further duodenal digestion (lane P3‐120, Figure 2A,B). These bands probably corresponded to the release of the protein fragments as well as protein subunits by trypsin hydrolysis, resulting in several bands not existing in the former profile. This result was similar to the previous study reported by Xing et al. (2017), in which additional bands were observed for soymilk curd with added tea polyphenols after simulated duodenal digestion. However, the bands in this study were fewer than those presented by Xing et al. during duodenal digestion, maybe due to the different material used, and chickpeas showed more sensitivity to trypsin hydrolysis than soy.

### 3.3 Bioaccessible Peptide

**FIGURE 3:**
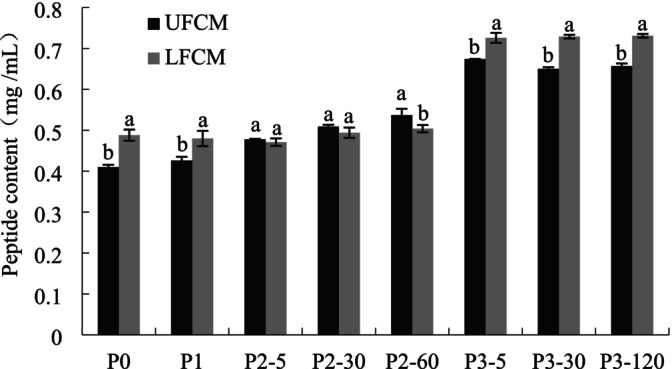
Peptide contents of UFCM and LFCM subjected to *in vitro* GIS. Within the same digestion phase, different lowercase letters indicate significant differences (*p* < 0.05). P0: Before the GIS; P1: After buccal digestion; P2‐5, P2‐30, P2‐60: Samples taken at 5 min, 30 min, and 60 min of gastric digestion; P3‐5, P3‐30, P3‐120: Samples taken at 5 min, 30 min, and 120 min of intestinal digestion. Data are expressed as mean ± SD of triplicates.

The results of bioaccessible peptides whose MM was lower than 10 kDa in the two samples are presented in Figure 3. Before digestion (P0), LFCM showed more peptide contents than UFCM (*p* < 0.05), which was probably due to the proteolytic effect of *L. plantarum* during the fermentation process. During the subsequent buccal digestion (P1) and gastric digestion (P2) process, the peptide contents increased slowly for UFCM by the hydrolytic action of pepsin but showed no obvious increase for LFCM (*p* > 0.05), this might be due to the lower soluble protein contents in LFCM in the P0 phase; thus, during the P1 and P2 phases, the trapped proteins in fermented chickpea milk had to be released to the solution first before they were further hydrolyzed into peptides. Peptide increased for both samples when entering the duodenal phase (P3), and the increase was larger (from 0.54 to 0.67 mg/mL for UFCM, 0.50–0.73 mg/mL for LFCM). These changes in peptide contents in the P3 phase reflected the SDS‐PAGE pattern of the two samples; SDS‐PAGE patterns revealed the disappearance of most bands in UFCM and LFCM gels when entering the duodenal digestion phase, suggesting that short peptides or amino acids were released, which were not retained by the gels. The observed peptide profile changes aligned with previous findings on fermented soymilk curds containing tea polyphenols (Xing et al. 2017), where *Weissella hellenica* D1501 fermentation at varying termination pH values similarly showed increasing peptide content during gastrointestinal (GIS) digestion. LFCM digests contained marginally higher peptide levels than UFCM after complete digestion, which indicated that more peptides were released from LFCM because of the presence of digestive enzymes in duodenal juices. The former study has demonstrated that peptides of smaller molecular weights, especially those composed of 2–10 amino acids, showed bioactivities (Carbonaro et al. 2015). Thus, after the *in vitro* GIS, both UFCM and LFCM would probably present more bioactivities due to the increase in peptide contents.

### 3.4 Free Amino Acid Analysis

**FIGURE 4:**
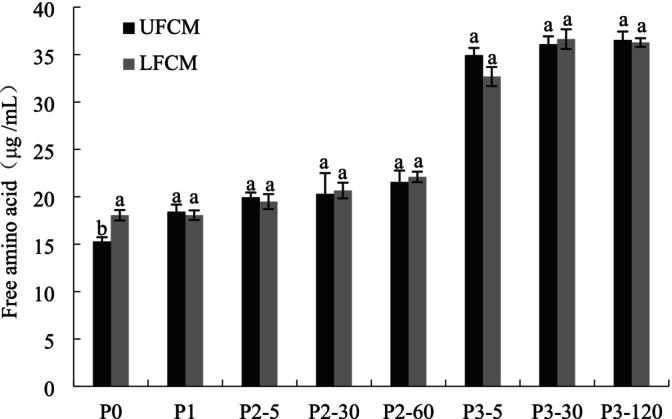
Free amino acids (FAAs) contents of UFCM and LFCM subjected to *in vitro* GIS. Within the same digestion phase, different lowercase letters indicate significant differences (*p* < 0.05). P0: Before the GIS; P1: After buccal digestion; P2‐5, P2‐30, P2‐60: Samples taken at 5 min, 30 min, and 60 min of gastric digestion; P3‐5, P3‐30, P3‐120: Samples taken at 5 min, 30 min, and 120 min of intestinal digestion. Data are expressed as mean ± SD of triplicates.

As shown in Figure 4, FAA content in both samples increased progressively during gastrointestinal (GIS) digestion, with the most significant rise occurring in the duodenal phase compared to the buccal and gastric phases. These findings align with Rinaldi et al. (2014), who observed minimal FAA release during buccal and gastric digestion but a marked increase during duodenal digestion across dairy matrices. The differential FAA production likely reflects the enzymatic composition at each stage: buccal and gastric juices lack peptidases, while duodenal juice contains endoproteases and peptidases that efficiently hydrolyze proteins into short peptides and FAAs. As seen in the former results of the peptide, little change occurred in the content of the peptide during the buccal and gastric digestions. At the end of digestion, there was a significant improvement in FAA contents (from 15.27 to 36.54 μg/mL for UFCM and 18.06–36.27 μg/mL for LFCM). The contents showed no significant difference between UFCM and LFCM digests (*p* > 0.05).

### 3.5 Peptide Analysis by HPLC

**FIGURE 5:**
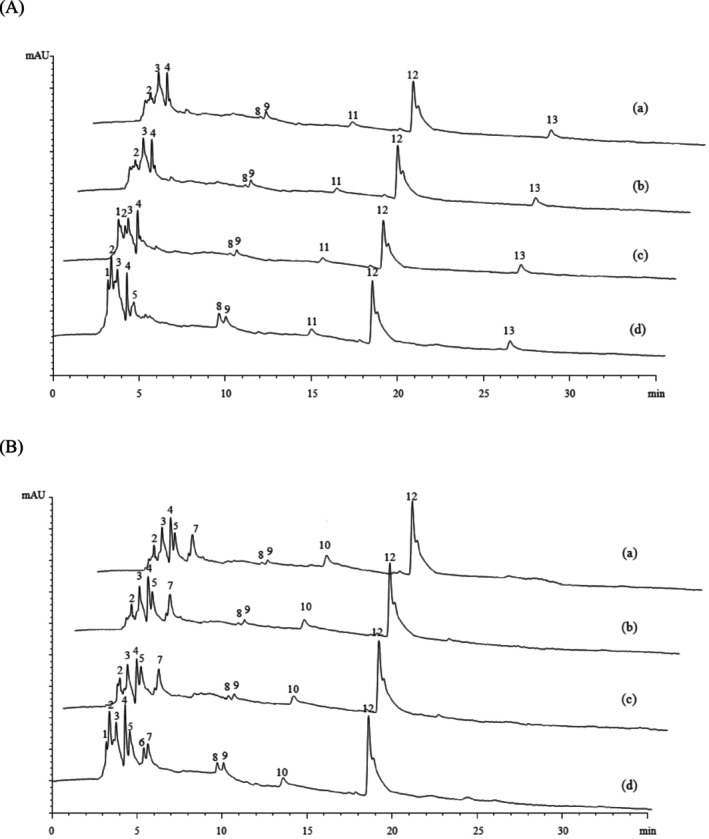
High‐performance liquid chromatography (HPLC) profiles of UFCM (A) and LFCM (B): (a) Before the GIS digestion (P0); (b) After buccal digestion (P1); (c) After gastric digestion (P2–60); and (d) After intestinal digestion (P3–120). Numbers 1–13 indicate the peaks selected for analysis.

To further illustrate peptide profiles of UFCM and LFCM during the digestion process, HPLC was conducted, and the results are shown in Figure 5. Thirteen peaks with different retention times (RT) were analyzed and named 1–13 (with RT from 3.185 to 26.60 min). The RT of small peptides seems to depend mainly on amino acid composition, while other factors like molecular weight, hydrophobicity, and conformational effects may influence the retention time of larger peptides too (Mant et al. 1988; Mant et al. 1989).

The profiles of UFCM and LFCM were similar, affording 8 and 9 major peaks, respectively, before digestion (Figure 5A(a), 5B(a)). Peaks 11 and 13 disappeared, and peaks 5, 7, and 10 appeared after fermentation. The differences in peaks may be attributed to the peptidase system of *L. plantarum*; thereby, proteins or peptides were hydrolyzed to form smaller peptides/amino acids during fermentation (Wu et al. 2015). HPLC profiles of both samples were almost unchanged after simulated buccal digestion (Figure 5A(b), 5B(b)), indicating that there was no proteolysis effect during the phase. Following digestion of UFCM and LFCM by pepsin, the area under many peaks changed (Figure 5A(c), 5B(c)). For UFCM, the number of peaks increased; peak 1 appeared, while peaks 3 and 13 decreased in size with decreases of 66% and 22%. Peaks 2, 9, and 11 increased in size with increases of 45%, 80%, and 59% by the end of gastric digestion. As for LFCM, after the gastric digestion phase, peaks 3, 9, and 12 decreased in size with decreases of 21%, 37%, and 18%. On the contrary, the area under peak 7 increased by 32%. HPLC analysis of pancreatin‐hydrolyzed peptic digests from both UFCM and LFCM yielded similar conclusions: the number of peaks further increased (Figure 5A(d), 5B(d)). For UFCM, one new peak, labeled as peak 5, appeared. For LFCM, two new peaks, labeled as peaks 1 and 6, appeared. Moreover, the area under more peaks increased, and the increase was greater than during the gastric digestion phase; typically, the areas under peaks 2, 8, and 9 increased by approximately 2.5‐, 1.4‐, and 1.4‐fold for UFCM, and increased by approximately 3.3‐, 1.8‐, and 1.5‐fold for LFCM. A few peaks decreased in size: peaks 11 and 13 for UFCM, and peak 7 for LFCM. The decreased peaks would be the consequence of the breakdown of peptides by digestive enzymes. As the digestive time increased, the peaks with short RT were higher and more intense than the undigested two samples, which indicated that more hydrophilic/smaller peptides were produced. These peptides might result from the protease and peptidolytic activities of the digestive enzyme, and the activity was higher during the duodenal digestion phase than the gastric digestion phase. There was also a reduction in the total hydrophobicity of the two samples since two highly hydrophilic chemical groups were released by breaking down every peptidic bond. Compared with UFCM, the LFCM profile showed more peaks with short RT by the end of GIS digestion. This was in agreement with previous studies, which reported that fermentation of milk by LAB contributed to the release of low molecular mass peptides during the digestive phase (Korhonen and Pihlanto 2006; Matar et al. 1996), also indicated that digestion after fermentation would generate more peptides than digestion or fermentation alone. Thus, more peptides were produced in the LFCM digest than UFCM, and this was further increased by fermentation before digestion.

### 3.6 Degree of Hydrolysis

**FIGURE 6:**
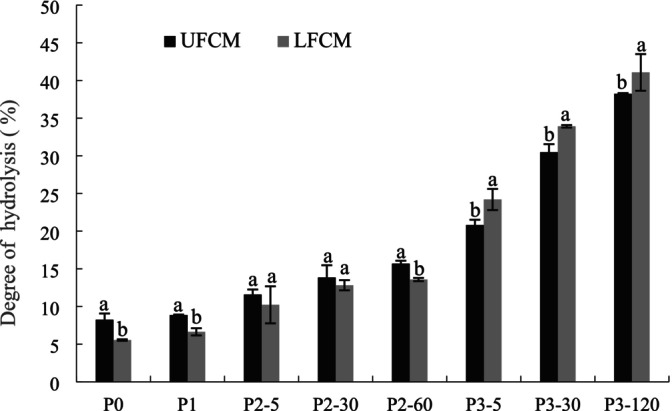
Degree of hydrolysis (DH) of UFCM and LFCM subjected to *in vitro* GIS. Within the same digestion phase, different lowercase letters indicate significant differences (*p* < 0.05). P0: Before the GIS; P1: After buccal digestion; P2‐5, P2‐30, P2‐60: Samples taken at 5 min, 30 min, and 60 min of gastric digestion; P3‐5, P3‐30, P3‐120: Samples taken at 5 min, 30 min, and 120 min of intestinal digestion. Data are expressed as mean ± SD of triplicates.

The degree of hydrolysis (DH) before digestion (P0), after 5 min of buccal digestion (P1), after 5, 30, and 60 min of gastric digestion (P2‐5, P2‐30, P2‐60), and after 5, 30, and 120 min of duodenal digestion (P3‐5, P3‐30, P3‐60) is presented in Figure 6. Both UFCM and LFCM showed an increase in DH during buccal digestion and gastric digestion; the DH rose from 8.25% to 15.69% for UFCM and from 5.55% to 13.58% for LFCM, which indicated chickpea protein of UFCM and LFCM was degraded by the action of pepsin. UFCM showed significantly higher digestibility (DH, *p* < 0.05) than LFCM prior to duodenal digestion, indicating greater resistance of LFCM to proteolytic enzymes. This reduced susceptibility likely stems from fermentation‐induced changes: lactic acid bacteria generate protons and acids that neutralize negative charges on chickpea proteins. When these particles reach zero net charge, gelation occurs, altering their enzymatic accessibility. Then the gel is linked by noncovalent bonds, including Vander Waals forces, hydrogen bonding (Ringgenberg et al. 2013), and hydrophobic interactions (Kohyama and Nishinari 1993), which may have caused lower susceptibility of chickpea protein to the proteolytic enzymes at the early stage of digestion. However, when entering the duodenal digestion phases, DH increased drastically, which demonstrated that more extensive proteolytic hydrolysis occurred through the action of pancreatin. At the end of duodenal digestion, LFCM showed higher DH (42.11%) than UFCM (38.23%); this could be due to the gradual release of trapped proteins, which became more sensitive to pancreatic fluid after LAB fermentation. Moreover, several oligopeptides produced by probiotic strain fermentation could generate peptides during subsequent digestion by pepsin and pancreatin. An increase in the quantity of peptides with low molecular mass may contribute to the increase in DH. The result was in accordance with that of peptide contents and peptide profile; LFCM showed more peptide contents and more peaks with short RT at the end of digestion.

### 3.7 Particle Size Distribution

**FIGURE 7:**
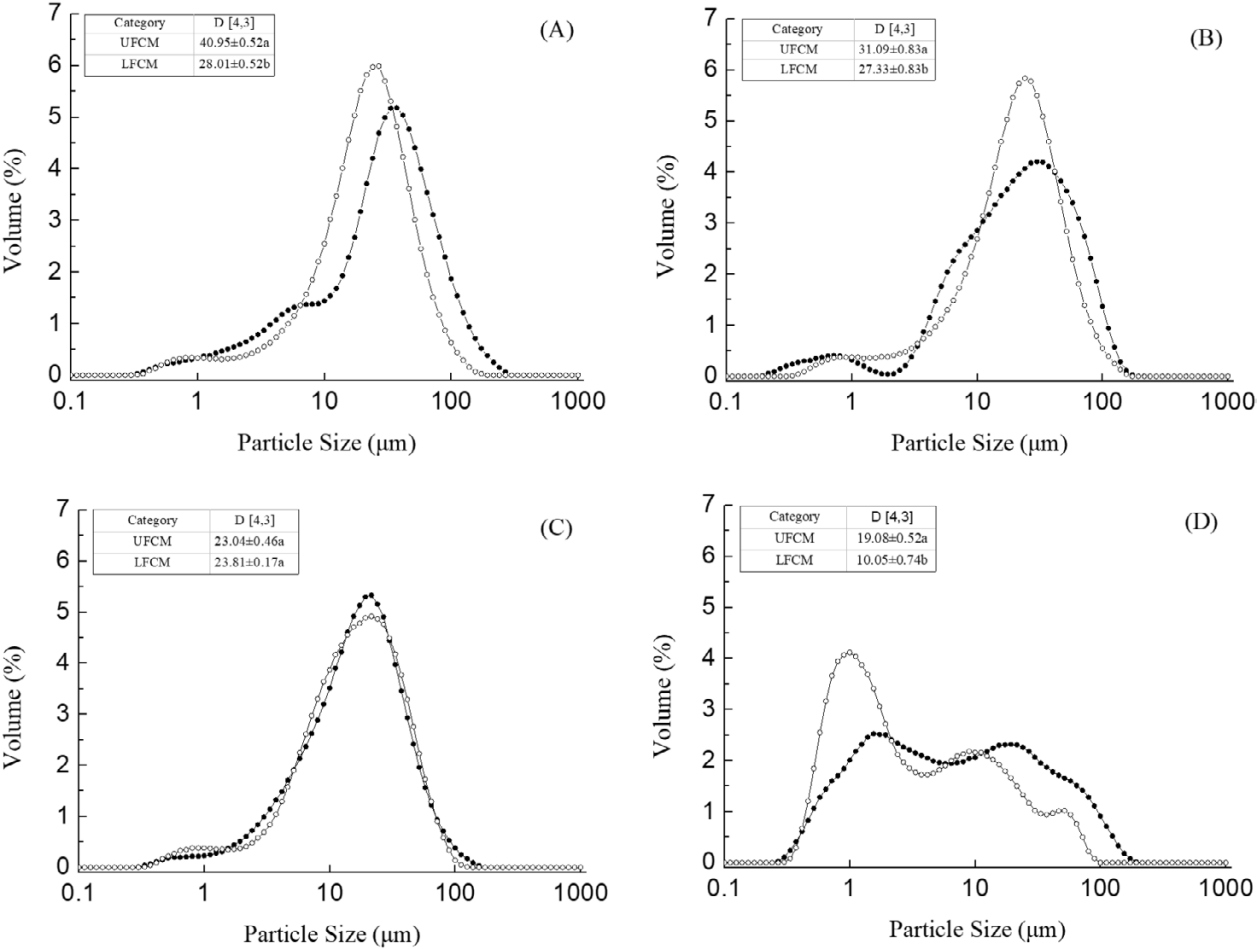
Particle size distribution of UFCM and LFCM subjected to in vitro GIS. (A) Before the GIS digestion (P0), (B) After buccal digestion (P1), (C) After gastric digestion (P2–60), and (D) After intestinal digestion (P3–120). Different patterns represent UFCM (black closed circle) and LFCM (black open square). Data are expressed as mean ± SD from triplicate experiments. Different letters within the same column indicate significant differences (*p* < 0.05).

The particle size distribution of UFCM and LFCM before and after *in vitro* gastrointestinal digestion is presented in Figure 7. Before digestion (P0), the mean particle size (D [4,3]) of UFCM was 40.95 ± 0.52 μm, while that of LFCM was smaller (28.01 ± 0.52 μm) than UFCM. This may be attributed to the effect of LAB fermentation; larger molecules were hydrolyzed during fermentation. For example, starch present in chickpeas ranges from 30.8% to 37.9% of the dry matter. Although high amylose content and resistant starch are resistant to enzymatic breakdown (Kaur and Prasad 2021), there is also digestible starch in chickpea milk, which could be broken down by lactic acid bacteria, which have potential amylolytic properties (Xu et al. 2019). There were no significant differences (*p* > 0.05) in D [4,3] of LFCM digest after buccal digestion (P1) compared with that in the P0 phase. However, UFCM showed a reduction of mean particle size (D [4,3]) by 24%. Perhaps the chickpea digestible starch of UFCM was hydrolyzed by α‐amylose during the buccal digestion (Grundy et al. 2016). As for LFCM, it was reported that after fermentation, higher values of resistant starch were found (De Pasquale et al. 2020), which could not be hydrolyzed or digested further. Moreover, perhaps high molecular weight chickpea protein fractions formed by acid gelation during lactic acid bacteria fermentation act as a physical barrier to prevent starch from being further hydrolyzed (Cordelino et al. 2019). The subsequent *in vitro* gastric digestion (P2) exhibited a disappearance of large particles for both samples; the D [4,3] value reduced to 23.04 ± 0.46 μm and 23.81 ± 0.17 μm for UFCM and LFCM, respectively. This could be related to extensive disruption of the chickpea protein network during gastric digestion by mechanical force, pepsin hydrolysis, and pH alteration (Rui, Xing, et al. 2016). Along with further digestion, larger chickpea protein particles were transformed into smaller ones, and a higher level of much smaller particles (< 1 μm) was generated as a result of the breakdown by pancreatic fluid and mechanical force. After 120 min of duodenal digestion, UFCM and LFCM digests exhibited a reduction of mean particle sizes (D [4,3]) by 17% and 58%, respectively. At the end of *in vitro* digestion, LFCM contained a smaller D [4,3], as indicated by 10.05 ± 0.74 for D [4,3]. This result further demonstrated that chickpea protein particles were easily broken down during *in vitro* gastrointestinal digestion and hydrolysis after lactic acid bacteria fermentation.

## 4 Conclusions

The present study investigated the protein bioaccessibility of unfermented chickpea milk (UFCM) and *L. planturum* fermented chickpea milk (LFCM) by an in vitro gastrointestinal digestion (GIS) model. The results obtained showed that the protein digestion during the in vitro GIS was different for the two samples, as expected. LFCM had lower soluble protein content, smaller mean particle size (D [4,3]), higher peptide quantity and degree of hydrolysis, and also presented slight differences concerning protein profiles and peptide profiles in comparison with chickpea milk after in vitro GIS. Overall, this study provided fundamental information about the potential protein bioaccessibility of LAB‐induced chickpea milk and demonstrated that fermentation of chickpea milk with LAB seemed to influence the proteins' behavior and interaction, thus interfering with the digestibility of chickpea proteins. A combination of digestion and fermentation could be more effective in chickpea protein bioaccessibility than digestion alone. Nevertheless, the in vitro GIS has certain limitations, including the absence of gut microbiota, nondynamic pH and flow conditions, and limited absorption and bioavailability data. Further studies are necessary to assess the protein bioaccessibility of LFCM under in vivo digestion conditions and determine whether its improved protein bioaccessibility enhances antioxidant activity and other bioactive effects in both in vitro and in vivo models.

## Conflicts of Interest

The authors declare no conflicts of interest.

## Data Availability

The data that support the findings of this study are available on request from the corresponding author. The data are not publicly available due to privacy or ethical restrictions.
